# Effects of variation in exercise training load on cognitive performances and neurotrophic biomarkers in patients with coronary artery disease

**DOI:** 10.1152/japplphysiol.00636.2023

**Published:** 2024-07-04

**Authors:** Maxime Boidin, Catherine-Alexandra Grégoire, Christine Gagnon, Nathalie Thorin-Trescases, Eric Thorin, Anil Nigam, Martin Juneau, Arthur Guillaume, Jonathan Tremblay, Mathieu Gayda, Louis Bherer

**Affiliations:** ^1^Research Center and Centre EPIC, Montreal Heart Institute, Montreal, Quebec, Canada; ^2^School of Kinesiology and Exercise Science, Faculty of Medicine, Université de Montréal, Montreal, Quebec, Canada; ^3^Department of Medicine, Faculty of Medicine, Université de Montréal, Montreal, Quebec, Canada; ^4^Department of Sport and Exercise Sciences, Institute of Sport, Manchester Metropolitan University, Manchester, United Kingdom; ^5^Liverpool Centre for Cardiovascular Science, Liverpool Heart and Chest Hospital, University of Liverpool, Liverpool John Moores University, Liverpool, United Kingdom; ^6^Centre de recherche, Institut Universitaire de Gériatrie de Montréal, Montreal, Quebec, Canada; ^7^Department of Surgery, Faculty of Medicine, Université de Montréal, Montreal, Quebec, Canada

**Keywords:** aerobic exercise, cognitive function, coronary artery disease, secondary prevention, training periodization

## Abstract

This study compared the effects of linear (LP) and nonlinear (NLP) training periodization on cognitive functions, neurotrophic biomarkers [plasma brain-derived neurotrophic factor (BDNF), insulin-like growth factor-1 (IGF-1)], and cathepsin-B in patients with coronary artery disease (CAD). Forty-four patients with CAD reported to our laboratory on two occasions to undergo testing procedures before and after training sessions, and were then blindly randomized to NLP or LP for 36 training sessions. *Visit 1* included blood samples and a maximal cardiopulmonary exercise testing to get maximal oxygen uptake (V̇o_2peak_). *Visit 2* included cognitive functions assessment. Thirty-nine patients completed the study (LP: *n* = 20, NLP: *n* = 19), with no observed changes in cognitive performances after the training intervention in either group. IGF-1 concentration decreased in both groups (time-effect: *P* < 0.001), whereas BDNF concentration increased (time-effect: *P* < 0.05) without group interaction, and cathepsin-B did not change after the intervention. Associations were found between ΔV̇o_2peak_ and ΔBDNF (*R*^2^ = 0.18, *P* = 0.04), and ΔIGF-1 and Δshort-term/working memory (*R*^2^ = 0.17, *P* = 0.01) in the pooled sample, with ΔIGF-1 and ΔBDNF accounting for 10% of the variance in Δshort-term/working memory. In the LP group, associations were found between ΔV̇o_2peak_ and ΔBDNF (*R*^2^ = 0.45, *P* = 0.02), ΔBDNF and Δshort-term/working memory (*R*^2^ = 0.62, *P* = 0.004), ΔIGF-1 and Δshort-term/working memory (*R*^2^ = 0.31, *P* = 0.01), and ΔIGF-1 and Δexecutive function (*R*^2^ = 0.22, *P* = 0.04). This study indicates that linear and nonlinear training periodization led to an increase in BDNF, and a decrease in IGF-1, without change in cognitive function in individuals with stable CAD.

**NEW & NOTEWORTHY** We used a novel and supervised iso-energetic training, integrating both moderate- and high-intensity aerobic exercises. Our findings indicate that greater variation in training load did not yield cognitive enhancements, although both protocols exhibited positive effects on brain-derived neurotrophic factor (BDNF) levels. Moreover, this study establishes a clear positive association between short-term and working memory and neurotrophic biomarkers. In addition, the independent predictive value of change in insulin-like growth factor-1 (IGF-1) on improvement in short-term and working memory highlight the close relationship between neurotrophic markers and cognition. Consequently, our results advocate for exercise training interventions targeting neurotrophic biomarkers to enhance cognitive function among individuals with coronary artery disease.

## Graphical abstract

**Figure:**
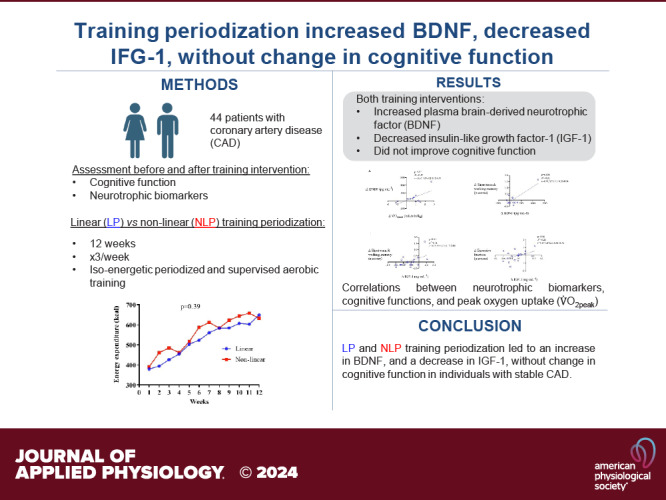

## INTRODUCTION

Cardiac diseases, which include coronary artery disease (CAD), are associated with an increased risk for cognitive impairment (1) and dementia (2). The prevalence of cognitive dysfunctions is estimated at ∼62% in individuals with CAD aged over 65 yr in the absence of stroke (3). Cognitive deficits in short-term, working, and long-term memories, executive functions, and processing speed have an impact on patient autonomy (4, 5). These cognitive impairments do not systematically lead to dementia, but could, for example, lead to reduced medication adherence and thus limit a patient’s ability to manage his/her own health care (4).

Possible underlying mechanisms linking CAD to cognitive dysfunctions include neurotrophic biomarkers such as brain-derived neurotrophic factor (BDNF), and insulin-like growth factor-1 (IGF-1). BDNF is known to facilitate neurogenesis, neuroprotection, and neuroregeneration (6), and reduced BDNF concentration is observed in patients with CAD (7), and may constitute a pathogenic factor common to Alzheimer’s disease (8). IGF-1 is the primary mediator of growth hormone effects, normally stimulates cardiac growth and contractile function, and is involved in the pathogenesis of CAD (9). As IGF-1 is essential for adult neurogenesis, its decrease with aging may contribute to cognitive decline (10). Cathepsin-B plays a role in intracellular proteolysis, is associated with an increased risk of cardiovascular events in patients with CAD (11), and has a neuroprotective role in Alzheimer’s disease (12). We recently showed that executive functions, processing speed, and BDNF concentration were impaired in physically active patients with CAD compared with healthy individuals (13). Recent reviews have highlighted the crucial need for a better exercise training intervention to improve cardiorespiratory fitness, and decrease psychological stress (and improve cognitive function) (14, 15). Thus, interventions that raise BDNF and IGF-1 levels are expected to become potential targets for the treatment of cognitive impairment and Alzheimer’s disease in the near future (16). Physical activity and aerobic exercise training have been shown to improve cognitive functions (17), increase BDNF (18) and IGF-1 (19, 20) concentrations in healthy elderly humans. However, despite high levels of cathepsin-B have been demonstrated in individuals with Alzheimer's disease (12), the role of exercise training on its level is less clear. A recent systematic review in a total of eight studies in young adults showed that three reported an increase in cathepsin-B with exercise, whereas one demonstrated a decrease, and three demonstrated no change. Half of the reported studies did not demonstrate change in cathepsin-B concentration after exercise training (21).

In a previous study, we showed that including more variation in an exercise training program (nonlinear periodization, NLP) did not lead to increased aerobic fitness (peak oxygen consumption, V̇o_2peak_) compared with convention training with linear progression (linear periodization, LP) in patients with CAD [PERIOD study: (22)]. Furthermore, improvements in cognitive performances and neurotrophic biomarkers are known to be independent of baseline V̇o_2peak_ (23, 24) but accompanied by changes in V̇o_2peak_ (25). As it is the case for V̇o_2peak_ improvement, alterations in neurotrophic factors and cognitive performances might depend on training factors including exercise training frequency, intensity, time, and type (FITT) (26–30). In a cohort of 217 individuals aged between 60 and 89 yr old, those in the highest tertile of physical activity intensity showed greater cognitive performances (29). The benefits of intensity and variation have been confirmed by other studies where higher levels of physical activity intensity (31–33) and more variation in exercises (34–36) have been associated with reduced risks of cognitive impairment. In rodents, a 3-mo aerobic training program raised BDNF levels (37), where BDNF progressively increased with longer training duration, regardless of how often exercise was performed (daily vs. every two days). Consequently, rather than aiming to increase V̇o_2peak_ to produce improvements in cognition and neurotrophic biomarkers, variation in FITT parameters could be a better approach in patients with CAD.

The present randomized controlled trial primarily aimed at comparing the effects of NLP versus LP on cognitive performances and neurotrophic biomarkers in patients with CAD. Second, we explored the association between changes in cognitive performances and neurotrophic biomarker concentrations. We hypothesized that NLP, which incorporates more training variation than LP, would lead to greater benefits on cognition and neurotrophic biomarkers as it has been observed in healthy individuals. Moreover, we hypothesized that changes in cognitive performances would be associated with an improved neurotrophic biomarker profile.

## METHODS

### Study Design and Patients’ Recruitment

The rationale and detailed study design have been published previously (22). Forty-four patients with CAD were recruited at the Cardiovascular Prevention and Rehabilitation Center (EPIC) of the Montreal Heart Institute from December 2015 to September 2017, and were blindly randomized to NLP or LP for 36 training sessions. The study protocol was approved by the Research Ethics and New Technology Development Committee of the Montreal Heart Institute and registered on ClinicalTrials.gov (Identifier Number: NCT03443193). All patients gave written consent before experimental testing.

### Inclusion and Exclusion Criteria

Inclusion criteria were as follows (22): *1*) age >18 yr old; *2*) documented CAD. Exclusion criteria were as follows: *1*) acute coronary syndrome <3 mo; *2*) heart failure; *3*) left ventricular ejection fraction (LVEF) <40%; *4*) severe CAD nonsuitable for revascularization; *5*) scheduled coronary artery bypass surgery for severe CAD; *6*) chronic atrial fibrillation; *7*) malignant arrhythmias during exercise; *8*) contraindication to cardiopulmonary exercise testing (CPET) or severe intolerance to exercise.

### Measurements

Before and after both periodized exercise training programs, all patients reported to our laboratory on two occasions to undergo morning testing procedures, separated by at least 24 h between visits. All patients were fasted without medications taken in the morning. During the first visit, all patients underwent a complete medical evaluation by a cardiologist that included medical history, blood draw, physical examination, anthropometric measures with body composition measurements (bioimpedance, Model BC418; Tanita Corporation of America, Arlington, IL), and a cardiopulmonary exercise testing (CPET). Cognitive performances were assessed during the second visit in all patients.

### Cardiopulmonary Exercise Testing

Patients completed an incremental CPET on a cycle ergometer (Ergoline 800S, Bitz, Germany) to exhaustion, following our previously reported protocol (22). Continuous electrocardiogram (ECG, Marquette, Case 12, St. Louis, MO), rating of perceived exertion (Borg Scale, 6–20), manual blood pressure using a sphygmomanometer (Welch Allyn Inc., Skaneateles Falls, NY), gas exchange, and power output were measured at each stage or every 2 min. Current medication was not interrupted before the CPET.

### Neuropsychologic Testing and Composite Scores

The neuropsychologic test battery included 11 cognitive tests to evaluate short-term and working memory, processing speed, executive functions, and long-term verbal memory by a certified neuropsychologist (38). All cognitive scores were first transformed into standardized *z*-scores. Composite scores were computed for processing speed (Digit Symbol Substitution Test, Trail Making Test [TMT] Part A, Stroop conditions 1 and 2), executive functions (TMT Part B, Stroop tasks 3 and4), and long-term verbal memory (immediate and delayed recall, and total words recalled during the five learning trials from the Rey Auditory Verbal Learning Test [RAVLT]). Digit span forward score was used to assess short-term memory and digit span backward for working memory (38). Cronbach αs were used to verify the internal consistency between all measures included in a composite score, considering a Cronbach alpha (α) > 0.7 to be acceptable (39). Results showed that each score had a valid reliability (short-term and working memory α = 0.773; processing speed α = 0.696; executive functions α = 0.717; long-term verbal memory α = 0.948).

### Neurotrophic Biomarkers

Blood samples were collected in EDTA K2-coated tubes, centrifuged to collect plasma. Plasma was kept at −80°C for 1 to 2 years before being analyzed in duplicates. Total IGF-1 (DG100, R&D Systems, Minneapolis, MN), BDNF (DBD00, R&D Systems), and cathepsin-B (b119584, Abcam, Cambridge, UK) plasma concentrations were measured by enzyme-linked immunosorbent assays (ELISA) in batched analyses using the frozen citrated plasma following the manufacturer’s instructions. These assays have inter- and intra-assay coefficients of variation of 8.5% and 4.5%, respectively. The laboratory member was blinded to patients’ group assignment when completing ELISA assay.

### Periodized Exercise Training Programs

Both training protocols were previously described (22). Briefly, all patients were scheduled for a supervised thrice-weekly periodized training session on a bicycle for 3 mo. Both LP and NLP were iso-energetic and included a total of 20 high-intensity interval (HIIT, duration: from 15 s to 4 min; intensity: between 80% and 100% of the peak power output) and 16 moderate-intensity continuous (MICT, duration: from 20 to 60 min; intensity: 50% to 70% of the peak power output) training sessions. Training load was increased by increasing the energy expenditure by an average of 5 ± 3% each week for 12 wk in the LP group, whereas in the NLP group, it was increased by an average of 8 ± 6% each week for 3 wk, followed by an average decrease of 4 ± 1% for 1 wk, and repeated for three cycles. Following the aerobic session, six different nonperiodized resistance exercises involving the main muscle groups, similar between the two groups, were performed by the participants.

### Physical Activity

Sedentary time and physical activity level were measured with the triaxial Actigraph GT3X+ (Actigraph Corporation, Pensacola, FL) accelerometer worn on the dominant hip, determined via the dominant hand, for seven days, 24 h. In addition, participants completed a diary recording any unusual additional activities, and any time they needed to remove the accelerometer. ActiLife software (v.6.13.3) was used to convert the 30-Hz ActiGraph.gt3.x files to raw 30.Hz csv files and counts in 1-s epochs with the low-frequency extension turned on. Data were cleaned and matched with the diary. Sedentary time, light (<3 metabolic equivalents, METs), moderate (3–<6 METs), and vigorous (≥6 METS) physical activities will be recorded (as well as moderate-to-vigorous physical activity, MVPA) (40).

### Statistical Analysis

Data are presented as means ± standard deviation (SD) or percentage unless otherwise specified. Differences were defined as statistically significant when *P* < 0.05 for a two-tailed test. Outliers were excluded when values were >3 SD (41). Baseline comparisons were performed using independent sample *t* tests or χ^2^ tests where appropriate. After ensuring a normal distribution, a two-way analysis of variance (ANOVA) with repeated measures (group × time) was used to compare cognitive performances and neurotrophic biomarker parameters between both groups. Bonferroni post hoc tests were used to detect differences when necessary. Effect size (Hedge’s *g*) was computed to evaluate the strength of the intervention between LP and NLP, by using the mean of the delta (post–pre) divided by the mean of the standard deviation of the delta. Pearson correlation analyses were also performed to detect possible correlations between changes (i.e., Δ) in V̇o_2peak_ and cognitive performances, and neurotrophic biomarkers, as well as between Δ in cognitive performances and Δ in neurotrophic biomarkers parameters in the pooled sample. To answer our research question whether variation in training load could impact cognitive performance, we replicated these analyses in the separated groups. The statistical analyses were performed in GraphPad Prism 9.3.1 (GraphPad Software, Inc., La Jolla, CA).

We also performed hierarchic linear regression analyses to verify whether changes in neurotrophic biomarkers were associated with changes in specific cognitive domains in the pooled sample and in the separated groups. First, we performed Pearson correlations between each neurotrophic biomarker and each cognitive domain. These correlations guided us for selecting variables to insert in the multiple linear analyses. Age, years of education, and sex were introduced in the first block of independent variables because these variables are known to be associated with cognitive performances (38). Then, additional parameters (Δneurotrophic biomarkers) were introduced in a second block to assess their relationships with the cognitive domains (42), based only on their significant correlations. For each block, the significance of the variation of *F* was considered to determine whether each set of independent variables explained a significant proportion of the variance. The coefficient of determination *R*^2^ was also used to quantify the proportion of the variable in the dependent variables (i.e., Δcognitive performances) that was predictable from the independent variables (i.e., Δneurotrophic biomarkers). The magnitude of the standardized β coefficient was also considered to understand the relative contribution of each independent variable in explaining our dependent variables.

## RESULTS

### Clinical Characteristics of the Patients

A total of 39 patients completed the study for the final analysis (LP: *n* = 20 and NLP: *n* = 19). Medication did not change during the training intervention. Body mass, body mass index (BMI), blood pressure, and heart rate did not change after each training intervention (Table 1).

**Table 1.** Clinical characteristics of the patients with coronary artery disease according to the training group (linear and nonlinear periodization)

	LP (*n* = 20)	NLP (*n* = 19)	*P* Value
Characteristics			
Sex (M/F)	15/5	14/5	0.93
Age, yr	65 ± 10	66 ± 5	0.55
Education, yr	15 ± 4	15 ± 3	0.80
Body mass, kg	83.3 ± 15.9	86.0 ± 17.0	0.59
BMI, kg·m^−2^	28.6 ± 4.7	29.5 ± 5.4	0.60
Fat mass, %	28.7 ± 8.9	30.1 ± 8.7	0.63
LBM, kg	58.9 ± 9.7	58.8 ± 11.5	0.98
Previous MI, *n* (%)	14 (70)	15 (79)	0.52
PCI, *n* (%)	12 (60)	14 (74)	0.36
CABG, *n* (%)	6 (30)	7 (37)	0.65
Smoker, *n* (%)	1 (5)	1 (5)	0.97
Hypertension, *n* (%)	12 (60)	10 (53)	0.64
Dyslipidemia, *n* (%)	15 (75)	16 (84)	0.48
Diabetes, *n* (%)	4 (20)	4 (21)	>0.99
Obesity, *n* (%)	5 (25)	6 (32)	0.65
Baseline V̇o_2peak_, mL·min^−1^·kg^−1^	22.0 ± 5.6	22.5 ± 5.2	0.79
Delta V̇o_2peak_, mL·min^−1^·kg^−1^	1.7 ± 1.9	1.2 ± 1.5	0.37
Baseline MVPA, min	25.3 ± 31.3	30.0 ± 20.3	0.59
Baseline sedentary time, min	707 ± 107	692 ± 100	0.66
Medication			
Aspirin, *n* (%)	20 (100)	17 (90)	0.14
DAPT, *n* (%)	11 (55)	12 (63)	0.60
RAAS inhibitors, *n* (%)	10 (50)	11 (58)	0.62
Beta-blockers, *n* (%)	11 (55)	12 (63)	0.60
CCB, *n* (%)	6 (30)	4 (21)	0.52
Diuretics, *n* (%)	2 (10)	1 (5)	0.58
Lipid lowering therapy, *n* (%)	20 (100)	19 (100)	>0.99
Antidiabetics (including insulin), *n* (%)	5 (25)	4 (21)	0.77
Blood sample parameters			
Total-cholesterol, mmol·L^−1^	3.7 ± 0.8	3.9 ± 1.1	0.68
LDL-cholesterol, mmol·L^−1^	1.7 ± 0.7	1.9 ± 1.0	0.59
HDL-cholesterol, mmol·L^−1^	1.4 ± 0.4	1.4 ± 0.3	0.69
Triglycerides, mmol·L^−1^	1.3 ± 0.6	1.4 ± 0.7	0.75
HbA1c, %	5.9 ± 0.6	6.0 ± 0.6	0.70

Continuous variables are expressed as means ± SD; dichotomous variables are expressed as numbers and percentages. BMI, body mass index; CABG, coronary artery bypass surgery; CCB, calcium channels blockers; DAPT, dual antiplatelet therapy; HbA1c, glycosylated hemoglobin; HDL, high-density lipoprotein; LBM, lean body mass; LDL, low-density lipoprotein; LP, linear periodization; MI, myocardial infarction; NLP, nonlinear periodization; PCI, percutaneous coronary intervention; RAAS inhibitors, inhibitor of the renin angiotensin aldosterone system including ARB (angiotensin receptor blockers) and ACE (angiotensin-converting enzyme); V̇O_2peak_, peak oxygen uptake.

### Cardiopulmonary Exercise Testing

As we previously reported (22), V̇o_2peak_ improved similarly in both groups following the 3-mo training program (LP: +5.3% and NLP: +8.1%, time-effect: *P* < 0.001). Adherence to training was similar in both groups and all patients performed 100% of the 36 supervised training sessions.

### Cognitive Performances, Neurotrophic Biomarkers, and Daily Physical Activity

No changes in cognitive performance measures were observed after the training intervention in either group (Table 2). IGF-1 decreased after the training intervention (time-effect: *P* < 0.001) without interaction. BDNF increased after the training intervention (time-effect: *P* < 0.05) without interaction. No change was observed for cathepsin-B. Moderate-to-vigorous physical activity, time at moderate intensity, and energy expenditure were decreased in both groups after training (time-effect: *P* = 0.02, *P* = 0.02, *P* = 0.007, respectively) without interaction. A trend is observed for an increase of sedentary time after the training intervention. Because physical activity is known to have an impact on cognition, MVPA and sedentary times were used as covariables. However, this did not change the results, and cognitive performances remained unchanged.

**Table 2.** Cognitive performances z-scores, neurotrophic biomarkers, and physical activity data in patients with coronary artery disease according to the training group (linear and nonlinear periodization)

	LP (*n* = 20)			NLP (*n* = 19)			ANOVA		
	Pre	Post	ES	Pre	Post	ES	Group Effect	Time Effect	Interaction
*Cognitive functions*									
MMSE									
Score	28.4 ± 1.2	28.7 ± 1.3	0.24	28.1 ± 1.3	28.5 ± 1.5	0.33	0.50	0.09	0.86
Proportion <28, *n* (%)	4 (20)	3 (15)		6 (32)	6 (32)				
MoCA									
Score	26.2 ± 3.1	25.9 ± 3.2	−0.15	26.4 ± 2.0	26.2 ± 2.1	−0.08	0.71	0.49	0.83
Proportion < 26, *n* (%)	5 (25)	8 (40)		5 (26)	6 (32)				
GDS	6.4 ± 5.4	6.4 ± 5.2	0.00	6.8 ± 6.5	6.5 ± 6.4	−0.09	0.90	0.79	0.79
Short-term and working memory	−0.12 ± 0.93	−0.06 ± 0.92	0.12	0.13 ± 0.9	0.06 ± 0.92	−0.15	0.52	0.94	0.41
Processing speed	0.00 ± 0.65	0.05 ± 0.93	0.08	0.00 ± 0.58	−0.06 ± 0.70	−0.22	0.80	0.98	0.51
Executive function	0.10 ± 0.76	0.06 ± 1.02	−0.06	0.01 ± 0.81	−0.18 ± 0.73	−0.58	0.51	0.17	0.36
Long-term verbal memory	−0.14 ± 0.99	0.00 ± 0.9	0.34	0.07 ± 0.90	0.08 ± 0.77	0.01	0.60	0.33	0.37
*Neurotrophic biomarkers*									
BDNF, pg·mL^−1^	221.4 ± 207.9	395.0 ± 491.6	0.48	352.4 ± 586.1	463.4 ± 631.4	0.38	0.60	**<0.05**	0.65
IGF-1, ng·mL^−1^	0.7 ± 0.2	0.5 ± 0.1	−1.08	0.7 ± 0.2	0.6 ± 0.2	−0.41	>0.99	**<0.001**	0.17
Cathepsin-B, ng·mL^−1^	238.6 ± 213.2	294.5 ± 439.4	−0.29	352.4 ± 463.4	586.1 ± 631.4	−0.11	0.50	0.25	0.69
*Daily physical activity*									
MVPA, min	25.3 ± 31.3	17.6 ± 13.2	−0.33	30.0 ± 20.3	21.8 ± 9.8	−0.52	0.46	**0.02**	0.93
Sedentary time, min	707 ± 107	760 ± 101	0.49	692 ± 100	702 ± 141	0.10	0.27	0.08	0.23
Time at light intensity, min	329 ± 95	341 ± 91	0.17	337 ± 106	344 ± 81	0.09	0.84	0.45	0.82
Time at moderate intensity, min	25 ± 31	17 ± 13	−0.33	30 ± 20	21 ± 10	−0.54	0.42	**0.02**	0.87
Time at vigorous intensity, min	1 ± 1	0 ± 1	−0.16	0 ± 0	0 ± 1	0.33	0.15	0.98	0.25
Steps (steps)	13,453 ± 5,824	14,209 ± 3,441	0.13	14,686 ± 5,125	13,988 ± 3,199	−0.20	0.70	0.97	0.36
Energy expenditure, kcal	429 ± 235	302 ± 160	−0.62	473 ± 238	367 ± 174	−0.37	0.32	**0.007**	0.79

Variables are expressed as means ± SD; dichotomous variables are expressed as numbers and percentages. BDNF, brain-derived neurotrophic factor; ES, effect size; GDS, Geriatric Depression Scale; IGF-1, insulin-growth factor-1; LP, linear periodization; MMSE, Mini-Mental State Examination; MoCA, Montreal Cognitive Assessment; MVPA, moderate-to-vigorous physical activity; NLP, nonlinear periodization. *P* values in bold when < 0.05.

### Associations between Changes in V̇o_2peak_, Neurotrophic Biomarkers, and Cognitive Performances

**Figure 1.**
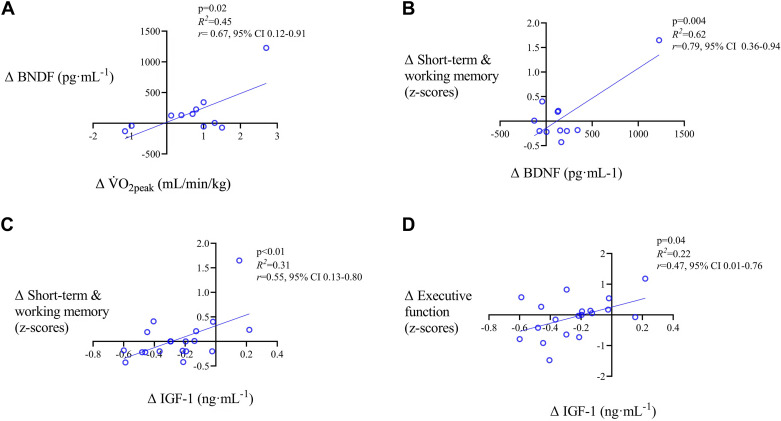
Correlation between changes in neurotrophic biomarkers, cognitive performances *z*-scores, and peak oxygen consumption (V̇o_2peak_) after the linear periodization training intervention. *A*: ΔV̇o_2peak_ and Δbrain-derived neurotrophic factor (BDNF). *B*: ΔBDNF and Δshort-term and working memory. *C*: Δinsulin-like growth factor-1 (IGF-1) and Δshort-term and working memory. *D*: ΔIGF-1 and Δexecutive function. Δ: change post-pre; CI, confidence interval.

When both intervention groups were combined, we found correlations between ΔV̇o_2peak_ and ΔBDNF (*R*^2^ = 0.18, *P* = 0.04) and a trend between ΔV̇o_2peak_ and Δcathepsin-B (*R*^2^ = 0.10, *P* = 0.06). Still in the pooled sample, no correlations were found between Δcognitive performances, and ΔV̇o_2peak,_ nor Δneurotrophic biomarkers, except between ΔIGF-1 and Δshort-term and working memory (*R*^2^ = 0.18, *P* = 0.04). When analyses were performed in the two training groups separately, correlations were found in the LP group only between ΔV̇o_2peak_ and ΔBDNF (*R*^2^ = 0.45, *P* = 0.02, Fig. 1*A*), between ΔBDNF and Δshort-term and working memory (*R*^2^ = 0.62, *P* = 0.004, Fig. 1*B*), between ΔIGF-1 and Δshort-term and working memory (*R*^2^ = 0.31, *P* = 0.01, Fig. 1*C*), and between ΔIGF-1 and Δexecutive functions (*R*^2^ = 0.22, *P* = 0.04, Fig. 1*D*). No correlations were found in the NLP group between neurotrophic biomarkers, V̇o_2peak_, and cognitive performances.

### Prediction Analyses

Based on significant correlation analyses, ΔBDNF and ΔIGF-1 were included in the prediction analyses for the pooled sample for Δshort-term and Δworking memory, Δprocessing speed, Δexecutive functions, and Δlong-term verbal memory. Besides sex, for short-term and working memory, prediction analyses showed that none of the traditional parameters (i.e., age, years of education) were significant predictors of cognitive performances in any domain (38). However, ΔIGF-1 (*P* = 0.04) and ΔBDNF (*P* = 0.07) together accounted for 10% of the variance in Δshort-term and working memory (Table 3). Prediction analyses remained similar even after adjusting for moderate-to-vigorous physical activity and total sedentary time.

**Table 3.** Regression analyses predicting changes in cognitive composite scores in patients with coronary artery disease

	*Model 1*			*Model 2*		
	*B*	β	*P* Value	*B*	β	*P* Value
*ΔShort-term and working memory*						
Sex	−0.292	−0.379	0.04	−0.323	−0.420	**0.02**
Age	0.016	0.321	0.11	0.008	0.166	0.41
Years of education	−0.021	−0.208	0.29	−0.031	−0.310	0.12
ΔBDNF				<0.001	0.351	0.07
ΔIGF-1				0.503	0.314	**0.04**
ΔCathepsin-B				<0.001	−0.074	0.64
*R* ^2^	0.30			0.40		
Δ*R*^2^	0.30			0.10		
Δ*F*	3.221		0.04	3.667		0.07
*ΔProcessing speed*						
Sex	−0.004	−0.044	0.85	−0.015	−0.169	0.50
Age	−0.018	−0.102	0.66	−0.033	−0.184	0.44
Years of education	−0.094	−0.069	0.74	−0.139	−0.102	0.63
ΔBDNF				0.001	0.285	0.22
ΔIGF-1				−0.040	−0.020	0.91
ΔCathepsin-B				<0.001	0.008	0.96
*R* ^2^	0.01			0.08		
Δ*R*^2^	0.01			0.07		
Δ*F*	0.091		0.96	1.581		0.22
*ΔExecutive functions*						
Sex	−0.005	−0.076	0.73	−0.003	−0.054	0.83
Age	0.037	0.286	0.20	0.039	0.301	0.21
Years of education	0.072	0.072	0.72	0.078	0.078	0.71
ΔBDNF				<0.001	−0.050	0.83
ΔIGF-1				0.448	0.240	0.16
ΔCathepsin-B				−0.001	−0.185	0.28
*R* ^2^	0.10			0.11		
Δ*R*^2^	0.10			0.01		
Δ*F*	0.883		0.46	0.050		0.83
*Long-term verbal memory*						
Sex	0.014	0.208	0.37	0.012	0.177	0.49
Age	0.018	0.127	0.58	0.015	0.107	0.66
Years of education	0.024	0.023	0.91	0.016	0.015	0.95
ΔBDNF				<0.001	0.070	0.76
ΔIGF-1				−0.077	−0.043	0.80
ΔCathepsin-B				<0.001	−0.023	0.89
*R* ^2^	0.04			0.04		
Δ*R*^2^	0.04			0.00		
Δ*F*	0.302		0.82	0.093		0.76

Δ, change post-pre; BDNF, brain-derived neurotrophic factor; CAD, coronary artery disease; IGF-1, insulin-growth factor-1. *P* values in bold when < 0.05.

## DISCUSSION

To the best of our knowledge, this study represents the first randomized controlled trial in patients with CAD comparing the effect of two periodized aerobic training protocols on cognitive functions, and neurotrophic biomarkers. It is also the first to explore whether changes in neurotrophic biomarkers are associated with changes in cognitive functions, and whether these parameters can predict changes in cognitive functions. First, we found that neither linear nor nonlinear training protocols improved cognitive functions in our population with stable CAD, but were similarly effective at increasing BDNF concentration. However, IGF-1 concentration was decreased after the 12-wk training intervention. Second, we found positive associations between ΔV̇o_2peak_ and ΔBDNF, ΔIGF-1 and Δshort-term and working memory in the pooled sample. When looking at both training groups separately, ΔV̇o_2peak_ was positively associated with ΔBDNF only in the LP group. Interestingly, ΔBDNF were associated with Δshort-term and working memory only in LP, but not in NLP. Similarly, ΔIGF-1 was associated with Δshort-term and working memory and Δexecutive functions only in LP, demonstrating that a decrease in IGF1 is associated with a decreased cognitive function after a LP intervention. These observations highlight the distinct effects of both periodized training protocols on the association between neurotrophic biomarkers and cognitive performances. Third, we found that ΔIGF-1 was an independent predictor of Δshort-term and working memory in the pooled sample. Taken together, these finding suggest that neurotrophic biomarkers might play an important role in cognitive changes with exercise training in patients with CAD. We also showed that more variation in training load did not lead to greater improvements in cognitive performances or neurotrophic biomarkers. LP seemed to have more impact on the association between changes in these neurotrophic biomarkers and performances in certain cognitive domains.

### Impact of Both Periodized Aerobic Training Protocols on Cognition and Neurotrophic Biomarkers

We recently demonstrated that physically active patients with stable CAD had impaired cognitive performances and reduced BDNF concentration compared with healthy individuals (13). However, the impact of exercise training could differ according to the population. A Cochrane systematic review of 754 cognitively healthy individuals taking part in aerobic training programs lasting from 8 wk to 26 wk reported no improvement in cognitive performances (43). Moreover, for those who improved their cardiorespiratory fitness (i.e., V̇o_2peak_), this improvement was not accompanied by any change in cognition, suggesting that improvement of the first does not necessarily lead to improvement of the second. Yet, there is a large variability regarding the exercise training prescription leading to heterogeneous results. Interestingly, based on a recent meta-analysis (44), only studies using a progressively increasing training load demonstrated an improvement of cognitive performances and neurotrophic biomarkers, whereas these parameters did not improve in studies where the training load changed little. These finding highlight the importance of increasing training load as is the case in training periodization (LP or NLP), to enhance cognitive and physiological adaptations during exercise training.

Surprisingly, only limited data exist on the effect of aerobic exercise training on cognitive functions in patients with CAD. Cardiac rehabilitation protocols incorporating a 12-wk exercise training program resulted in improved cognitive performances according to two studies (45, 46). However, several limitations must be noted. First, the cognitive test battery was different between studies and limited to five tests. Second, there was no measure of physical activity performed outside of supervised exercise sessions. Third, patients with heart failure were included in the cardiac rehabilitation programs. These patients are known to have worse cognitive impairment compared with patients with CAD alone (47). In our study, we used an extended range of cognitive tests to better capture the specificity of cognitive functions. Moreover, we used two different but supervised, periodized, iso-energetic aerobic exercise training programs that followed the current guidelines (48). Only patients with CAD took part in our study. Taken together, data from our randomized controlled trial suggest that a 3-mo periodized (LP and NLP) aerobic exercise training program did not improve cognitive performances in a homogenous population with CAD. This lack of improvement in cognitive functions cannot be totally explained by the fact that our participants were cognitively healthy at recruitment [mean Montreal Cognitive Assessment (MoCA) > 26 and mean Mini-Mental State Examination (MMSE) > 28] (13). The main reason is that the proportion of MoCA > 26 and MMSE > 28, even if statistically similar between the two groups, before and after training (all between groups χ^2^ test *P* values > 0.05), was between 15 and 40%. Finally, since greater improvement in V̇o_2peak_ might be related to greater improvement in cognitive performances (25), the modest changes in V̇o_2peak_ in our study could limit the benefits on cognition. As explained in our previous study (22), this modest increase in V̇o_2peak_ could be explained to a high V̇o_2peak_ baseline, which is considered a predictor of nonresponse to V̇o_2_ improvement in patients with CAD (49).

We investigated the exercise training-related changes in neurotrophic biomarkers, where only BDNF was similarly improved following both periodized training interventions. Our results are partially in line with the literature (44). For example, a meta-analysis involving 514 individuals with mild cognitive impairment (MCI) demonstrated that physical activity led to increased BDNF and IGF-1 concentrations (50). However, although BDNF increased in our study, IGF-1 decreased after the training intervention. The aforementioned meta-analysis combined resistance and aerobic exercise, making their conclusion scattered, as suggested by another review (51). That is, resistance exercise training seems to have greater effects on IGF-1 compared with aerobic exercise training in older adults (52), patients with MCI (53) and dementia (54). An interesting randomized controlled trial compared the effect of 16 wk of aerobic to resistance exercise training in 55 older adults with MCI (53); both aerobic and resistance exercise interventions improved cognitive functions to a greater extent than a control group without exercise training. BDNF increased after both training interventions, but IGF-1 increased only in the resistance training group, whereas solely aerobic training led to a decrease of some inflammatory cytokines such as tumor necrosis factor alpha (TNF-α) and interleukin-5 (IL-5). This study demonstrated that both training modalities resulted in improvement in cognitive functions, but probably through different mechanisms. Based on the current guidelines for patients with CAD (48), our training prescription included a periodized aerobic training (LP or NLP) and a similar nonperiodized resistance training program. This program involved the main muscle groups and lasted ≈20 min per session. Our intervention might have been too short compared with the previous study (12 vs. 16 wk), and the resistance training sessions per se might have been too short to impact neurotrophic biomarkers and cognitive performances in our study. The light resistance training program in our study might be responsible for the decrease in IGF-1. In addition, the duration of our training intervention might be too small (12 wk), since a longer intervention (>24 wk) seems to have more benefits on IGF-1 (51). Finally, MVPA has decreased in both groups in our study and could explain the decrease in IGF-1 after the 12-wk intervention. Our study did not aim at comparing resistance to aerobic exercise training, but two different aerobic training periodizations. Further studies comparing aerobic to resistance training on cognition and neurotrophic biomarkers in patients with CAD are needed.

### Association between Cognition, Neurotrophic Biomarkers, and Exercise

Even though there are several animal and human studies that demonstrated the association between cognitive functions, neurotrophic biomarkers, and exercise training (55), our study is the first that investigated this association in patients with CAD. Yet, these patients are known to be at greater risk of cognitive impairment compared with age-matched controls (1, 3). Maass et al. (56) performed an interesting study investigating the relationship between these three parameters in addition to changes in hippocampal volume, perfusion via magnetic resonance imaging (MRI), and memory in sedentary healthy older adults (*n* = 40, 68.4 ± 4.3 yr old, 55% females). Authors demonstrated that first, a 3-mo MICT did not change neurotrophic biomarkers, despite an improvement of V̇o_2peak_. Second, aerobic fitness-related benefits in hippocampal perfusion and volume were not associated with changes of any of the aforementioned neurotrophic biomarkers, suggesting that the changes in vascular hippocampal plasticity and neurotrophic biomarkers could be partially explained by other mechanisms. Third, these aerobic fitness-related benefits in the aged hippocampus were closely associated with positive Δmemory performances. Finally, ΔIGF-1 was positively correlated with hippocampal volume, independently of ΔV̇o_2peak_.

In the pooled samples of the present study, we found a positive association between ΔBDNF and Δshort-term and working memory. In the pooled samples and following LP alone, we also found that a decrease in IGF-1 was associated with a decrease in short-term and working memory and executive functions performances. These associations demonstrate that although neither training protocol led to cognitive improvement overall, individual changes may still be observed. This aligns with recent studies investigating variability in exercise response (57–59). This individual response to exercise training is mainly explained by genetics and environmental factors, and could explain the intervariability in our study. As with IGF-1, greater exercise-related increases in BDNF concentration were also associated with increased hippocampal volume (17). Moreover, BDNF has been considered as a mediator of improvements in executive functions in the elderly (*n* = 90, 67 yr old) (60). In fact, BDNF is also found in the prefrontal cortex that likely supports executive functions (61). Interestingly, the moderate effects of acute aerobic exercise are potentiated when it is performed repeatedly as part of a chronic exercise regimen, leading to amplified outcomes and greater benefits (28). In this meta-analysis, authors suggested that each acute aerobic exercise results in a dose of BDNF activity and that the magnitude of this dose can be increased over time by regular exercise. The same explanation might be observed for IGF-1. In our study, training load was constantly increased in the LP group, while it was intercepted by recovery weeks (i.e., decrease in training load) in the NLP group. Thus, incorporating a recovery week in NLP could limit the ΔBDNF, whereas the constant increase in the training load in LP could be a potential explanation why ΔBDNF was associated with Δcognitive performances, and why a decreased IGF-1 was associated with a decreased cognitive performances. Another possible explanation might be the variation of the training load. Even if both training protocols were iso-energetic in our study, the difference in the training load variation may lead to different exercise-induced associations between Δneurotrophic biomarkers and Δcognitive performances. Animal (62) and human (60) studies reported that some aspects of the FITT principles [training volume (62) and duration (60)] have distinct impacts on ΔBDNF, and that could be another speculative explanation of the specific associations in LP.

Another novelty of our study is that ΔIGF-1 positively predicts Δshort-term and working memory in patients with CAD. These findings are consistent with the literature, where these cognitive domains are reported to be more sensitive to training adaptations (63). These findings clearly demonstrate the close relationship between neurotrophic biomarkers and cognition, and the impact of exercise training in patients with CAD.

### Strengths and Limitations

Our study has several strengths, which include the randomized controlled design, the use of an extended range of cognitive tests to better represent the specificity of cognitive functions, and the novel supervised, personalized, periodized aerobic exercise training prescription. However, one important limitation relates to the lack of neuroimaging data limiting the understanding of the underlying mechanisms. We followed the recommendations in patients with CAD regarding the training prescription and included resistance training in both groups. Considering that resistance training could also have positive impacts on cognition, it is difficult to understand the distinct effect of the aerobic component on cognition. Another limitation is the recruitment of patients only without cognitive impairment (MoCA > 26, MMSE > 28), and with high baseline aerobic fitness (mean of 102% and 106% of the predicted values for healthy individuals in LP and NLP, respectively). The decrease of physical activity level between pre- and postmeasurements might be explained by the period when data have been collected. Our center is based in Canada where Summer can be warm and Winter cold with snow. Premeasurement have mainly been collected during Summer/Autumn time while postmeasurements have been collected during Winter time. This could impact physical activity, mainly in older population. However, we included MVPA and sedentary times in our prediction analyses. Finally, a major limitation is the lack of control group, and therefore it cannot be determined whether improvements in neurotrophic biomarkers following both of our training protocols would occur naturally without exercise training. Further studies with patients with more severe cardiac diseases (e.g., heart failure), cognitive dysfunctions, and the addition of neuroimaging will be needed to explore the impact of exercise training on cognition.

In conclusion, cognitive performances of patients with CAD were neither improved by linear nor nonlinear periodized exercise training. However, circulating levels of BDNF increased after the 3-mo interventions. Moreover, changes in short-term and working memory were positively associated with changes in BDNF, and a decrease in IGF-1 was associated with a decrease in cognitive performances, suggesting that changes in some cognitive domains are related to variation in neurotrophic biomarkers. Finally, we demonstrated that the decrease in IGF-1 concentration after the intervention was considered an independent predictor for a decrease in short-term and working memory performances, highlighting the impact of this neurotrophic biomarker on specific domains of cognition. Since cardiac and cognitive functions are interconnected (64), future studies are needed to further understand how exercise training impacts this Heart-Brain Axis.

## DATA AVAILABILITY

The data that support the findings of this study are available from the corresponding author upon reasonable request.

## GRANTS

This study was financially supported by the Montreal Heart Institute and the EPIC Center Foundations. M.B. is financially supported by a grant from the Fonds de Recherche du Québec-Santé (FRQ-S).

## DISCLOSURES

No conflicts of interest, financial or otherwise, are declared by the authors.

## AUTHOR CONTRIBUTIONS

M.G., A.N., and M.J. conceived and designed research; M.G. performed experiments; M.B. analyzed data; M.B., C.-A.G., C.G., N.-T.T., E.T., A.N., M.J., A.G., J.T., M.G., and L.B. interpreted results of experiments; M.B. prepared figures; M.B. drafted manuscript; M.B., C.-A.G., C.G., N.-T.T., E.T., A.N., M.J., A.G., J.T., M.G., and L.B. edited and revised manuscript; M.B., C.-A.G., C.G., N.-T.T., E.T., A.N., M.J., A.G., J.T., M.G., and L.B. approved final version of manuscript.
